# Treatment With 2-BFI Attenuated Spinal Cord Injury by Inhibiting Oxidative Stress and Neuronal Apoptosis *via* the Nrf2 Signaling Pathway

**DOI:** 10.3389/fncel.2019.00567

**Published:** 2019-12-20

**Authors:** Xiaolong Lin, Jie Zhu, Haibo Ni, Qin Rui, Weiping Sha, Huilin Yang, Di Li, Gang Chen

**Affiliations:** ^1^Department of Orthopaedic Surgery, The First Affiliated Hospital of Soochow University, Suzhou, China; ^2^Department of Orthopaedic Surgery, The Affiliated Zhangjiagang Hospital of Soochow University, Zhangjiagang, China; ^3^Department of Anesthesiology, The Affiliated Zhangjiagang Hospital of Soochow University, Zhangjiagang, China; ^4^Department of Neurosurgery, The Affiliated Zhangjiagang Hospital of Soochow University, Zhangjiagang, China; ^5^Department of Laboratory, The Affiliated Zhangjiagang Hospital of Soochow University, Zhangjiagang, China; ^6^Department of Translational Medicine Center, The Affiliated Zhangjiagang Hospital of Soochow University, Zhangjiagang, China; ^7^Department of Neurosurgery and Brain and Nerve Research Laboratory, The First Affiliated Hospital of Soochow University, Suzhou, China

**Keywords:** 2-BFI, Nrf2, HO-1, spinal cord injury, neuroprotection

## Abstract

Previous reports showed that 2-(-2-benzofuranyl)-2-imidazoline (2-BFI) has antioxidant, anti-inflammatory and anti-apoptotic effects on neuroprotection in numerous disorders. However, the precise mechanisms remain elusive. The nuclear factor c factor 2 (Nrf2)/antioxidant response element (ARE) signaling pathway plays an important and essential role in the antioxidant and anti-inflammatory responses of the cell. Therefore, the purpose of this study was to investigate the potential neuroprotective effects of 2-BFI in a rat model of spinal cord injury (SCI) and to determine whether its neuroprotective effects are associated with the activation of Nrf2. To test this hypothesis, we examined the potential roles of 2-BFI in SCI models which were established in rats using a clip-compression injury method. Our results showed that treatment with 2-BFI twice daily improved locomotion recovery from SCI, which increased Nrf2 expression in both neurons and astrocytes, meanwhile, the level of heme oxygenase-1 (HO-1) also significantly enhanced. In addition, after the treatment with 2-BFI increased levels of superoxidase dismutase (SOD) and glutathione peroxidase (GPx) indicated the antioxidant effect of the drug. Furthermore, the upregulation of Bcl-2 and downregulation of Bax and caspase-3 implied antiapoptotic effects on neuroprotection of 2-BFI, which were verified by the Fluoro-Jade B (FJB) staining and TUNEL staining. Collectively, these results add to a growing body of evidence supporting that 2-BFI may attenuate SCI mediated by activation of the Nrf2/HO-1 signaling pathway.

## Introduction

Spinal cord injury (SCI) is one of the most serious conditions that cause limb and trunk dysfunction in countries worldwide. Over 1 million patients live with an SCI, and more than 12,000 new cases of SCI are reported each year in the United States (Hachem et al., 2017). SCI involves biphasic pathophysiological stages that influence the severity of dysfunction. Although the pathophysiology of SCI has been continuously investigated, the precise pathogenesis has not yet been fully elucidated. Several studies revealed the pathophysiology of injury, including neuronal inflammation, reactive changes in the glia, oxidative stress, neuronal degeneration and apoptosis (Chen et al., 2018; Lu et al., 2018; Meng et al., 2018). Disturbances in the spinal cord blood flow (SCBF), severely reduced oxygen levels, perfusion defects, hemorrhage, ischemia, and hypoxia have been demonstrated as a focus of post-injury pathophysiological changes of acute SCI (Huo et al., 2019).

Imidazoline receptors (IRs) originally referred to the binding sites that are recognized by radiolabeled adrenergic ligands, are insensitive to norepinephrine and are not adrenergic receptors (Li, 2017). At least two different subtypes called imidazoline I_1_ and I_2_ receptors were identified (Li, 2017). Accumulating studies have shown that 2-(2-benzofuranyl)-2-imidazoline (2-BFI), a selective ligand to type 2 IRs, has prominent neuroprotective effects in animal models of many disorders, such as traumatic brain injury, stroke, cerebral ischemia, and Alzheimer’s Disease, even in some nociception (Han et al., 2009, 2010; Jiang et al., 2010; Sampson et al., 2012; Tian et al., 2017; Ni et al., 2019). Moreover, a recent study found that 2-BFI effectively protected against SCI caused by experimental autoimmune encephalomyelitis, a mouse model of multiple sclerosis (Wang et al., 2011).

Activation of Nrf2, a sensor of oxidative stress, played neuroprotective roles in animal models of cerebral ischemia, subarachnoid hemorrhage, traumatic brain injury and SCI (Wang et al., 2014). Nrf2 regulates a number of ARE-driven genes encoding phase II detoxification enzymes and antioxidant proteins like HO-1 (Niture et al., 2010). Heme oxygenase-1 (HO-1) is known as an endogenous antioxidative enzyme in the injured spinal cord. Several studies have shown that HO-1 is sustained in traumatic SCI (Lin et al., 2016, 2017; Lee et al., 2017). Furthermore, HO-1 was found to stabilize the blood-spinal cord barrier and limit oxidative stress and white matter damage in the acutely injured murine spinal cord (Lin et al., 2007).

These findings led us to hypothesize that 2-BFI could attenuate SCI mediated by activation of the Nrf2/HO-1 signaling pathway. However, no previous studies focused on the neuroprotective effect of 2-BFI on SCI. Thus, in our study, we aimed to investigate whether 2-BFI played a neuroprotective role in an SCI rat model and to further explore the potential mechanism.

## Materials and Methods

### Experimental Animals

We included 52 adult male Sprague–Dawley (SD) rats (290–330 g) in this study that were obtained from the Animal Center of the Chinese Academy of Sciences (Shanghai, China). The rats were housed in a humidity-controlled room (25 ± 1°C, 12-h light/dark cycle, with lights on at 7:00 am) and raised with free access to food and tap water. All experimental protocols were approved by the Animal Care and Use Committee of Soochow University and were implemented with reference to the Animal Research: Reporting of *in vivo* Experiments (ARRIVE) guidelines (Permit Number: 2018-0006). After being acclimatized for 2 weeks, the rats were randomly divided into the following three groups with no labels (*n* = 48).

### SCI Model

The experimental SCI model was established as described previously (Soubeyrand et al., 2014; Can et al., 2017) and we selected two of three groups randomly undergoing the SCI surgery. Briefly, general anesthesia was achieved by administration of 5% isoflurane and maintenance by 1.5% isoflurane. After a deep level of anesthesia, the rat was placed in a prone position on a surgical table. The dorsal operation area of each rat was shaved subsequently local antisepsis was performed. Animals received incision analgesia with lidocaine. The spinous process of the T10 thoracic vertebra was located by palpating the ribs and double confirmed with the dorsal process of the 2nd thoracic (Th2) vertebra, the most reliable anatomic landmark in rats (the tallest vertebra in rats when placed in the prone position). A dorsal midline incision was made, and a laminectomy was performed from the 10th thoracic (Th10) vertebra to the 12th thoracic vertebra (Th12) until the dorsal epidural surface of the spinal cord was completely exposed. Rats in the SCI+vehicle group and SCI+2-BFI group received compression by an aneurysm clip, while in the remaining rats, the Th10–Th12 laminas were removed until the spinal cord was exposed without causing SCI (the sham group). The spinal cord of the rats was compressed for 60 s using a 70-g closing force Yasargil aneurysm clip (FT 220T, B. Braun Meslungen AG, Melsungen, Germany). Indicators of successful injury included the red and swollen appearance of the local spinal cord, fluttering of both hindlimbs immediately after compression, and bilateral hindlimb paralysis when the rats were awake. After the clip was removed, the incisions were closed with silk thread, and the animals were allowed to recover in a warmed chamber before being returned to their home cages. The urinary bladder was manually emptied twice daily to assist in urination until the micturition function returned to normal.

### Drug Administration

**Figure 1:**
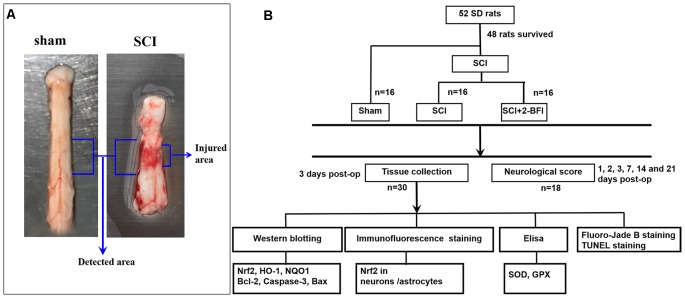
Spinal cord tissue surrounding the damaged site was immediately collected after the animals were deeply anesthetized **(A)**. Experimental design: experiments were designed to show the potential neuroprotective effects of 2-BFI in spinal cord injury (SCI; **B**).

Drug administration was carried on by an investigator who was blind to the drugs. After that, the rats were divided into three groups: (1) sham group; (2) SCI+vehicle group; and (3) SCI+2-BFI group (Figure 1B). Rats in the SCI+2-BFI groups received an intraperitoneal injection of 2-BFI (SML1703, Sigma-Aldrich, St. Louis, MI, USA) at 3 mg/kg twice daily according to previous research (Tian et al., 2017). Meanwhile, rats in the sham and SCI groups received 0.9% NaCl twice daily instead.

### Locomotion Tests

Six rats of each group were randomly selected to perform a locomotion recovery test on day 1, 2, 3, 7, 14, 21 days after the operation. The locomotion was assessed using the Basso, Beattie and Bresnahan (BBB) locomotor rating scale, which is used to assign scores ranging from 0 points to 21 points (Basso et al., 1995, 2006). A score of 21 points refers to normal locomotion, and 0 points indicate complete paralysis. Each rat was evaluated three times by a highly trained investigator who was unaware of the experimental design, and the mean value of the scores was taken.

### Tissue Collection

After the treatment of 2-BFI twice daily, the rats were deeply anesthetized with intraperitoneal sodium pentobarbital (100 mg/kg) at day 3 after the operation. A 1.5 cm section of spinal cord tissue surrounding the damaged site was immediately collected on ice and perfused with 200 ml of 4°C 0.9% saline (Figure 1A). For the chemical evaluation, the obtained tissue samples were rapidly transferred to liquid nitrogen and stored at −80°C for further use. For the histopathological analysis, the obtained tissue samples were immersed in 4% paraformaldehyde at 4°C overnight and cryoprotected in a 20% sucrose solution and 30% sucrose solution for 24 h in turn. All frozen spinal cord sections were cut at a thickness of 10 μm by using a sliding microtome. Systematic random sampling techniques were used to select tissue sections for staining and stereological analysis. Every fifth section beginning at a random start point was selected for the appropriate staining procedure. All processes used for tissue resection and selection were conducted by two pathologists who were blinded to the experimental conditions.

### Western Blotting

The damaged spinal cord tissues were removed at the center of the injury extending 5-mm cephalad and caudally. Immediately, the removed tissues were homogenized and lysed in ice-cold RIPA buffer (P0013B, Beyotime Biotechnology, China) supplemented with protease and phosphatase inhibitor cocktails (ab201119, Abcam, Burlingame, CA, USA). The homogenates were centrifuged at 12,000 rpm for 20 min at 4°C, and then, the supernatant was collected on ice. Then, the final protein concentration was determined by using the bicinchoninic acid (BCA) method with the Pierce™ BCA Protein Assay Kit (23225, Thermo Fisher Scientific, Waltham, MA, USA). Equal amounts of protein were loaded and separated on sodium dodecyl sulfate-polyacrylamide gels (SDS-PAGE). Subsequently, the separated proteins were transferred onto PVDF membranes (IPVH00010, Millipore, Burlington, MA, USA) and blocked with 5% nonfat milk powder in Tris-buffered saline-Tween (TBST, 25 mM Tris-HCl, 0.15 M saline and 1% Tween 20; CW0043S, CWBIO, China) for 2 h at room temperature, and the PVDF membranes were incubated overnight at 4°C with the following primary antibodies: anti-Nrf2 antibody (1:1,000, ab137550, Abcam, Burlingame, CA, USA), anti-Heme Oxygenase 1 antibody (1:2,000, ab13243, Abcam, Burlingame, CA, USA), anti-NQO1 antibody (1:1,000, ab34173, Abcam, Burlingame, CA, USA), anti-Bcl-2 antibody (1:1,000, ab59348, Abcam, Burlingame, CA, USA), BAX rabbit polyclonal antibody (1:2,000, 50599-2-Ig, Proteintech, Rosemont, IL, USA), and Caspase 3 rabbit polyclonal antibody (1:500, 19677-1-AP, Proteintech, Rosemont, IL, USA). Monoclonal anti-β-actin antibody (1:10,000, A5316, Sigma-Aldrich, St. Louis, MI, USA) was used as an internal loading control. The next day, after being washed with TBST buffer three times, the membranes were incubated with the following horseradish peroxidase-conjugated secondary antibodies for 2 h at room temperature: goat anti-rabbit IgG secondary antibody, HRP (31431, Invitrogen, Carlsbad, CA, USA) and goat anti-mouse IgG secondary antibody, HRP (31430, Invitrogen, Carlsbad, CA, USA). After being washed with TBST three times, immunoreactive bands were finally enhanced by an Immobilon™ Western Chemiluminescent HRP Substrate (WBKLS0500, Millipore, Burlington, MA, USA) and visualized with an imaging system (Bio-Rad, USA). Finally, all data were analyzed by grayscale using ImageJ software.

### Immunofluorescence Staining

After being fixed with a 4% formaldehyde solution, frozen spinal cord sections (10 μm) were washed with phosphate-buffered saline (PBS; 21-040-CVR, Corning, USA) with 0.1% Triton X-100 for 30 min and then blocked with 10% goat serum for 1 h at room temperature. Then, the sections were incubated at 4°C overnight with primary antibodies as follows: anti-Nrf2 antibody (1:200, ab137550, Abcam, Burlingame, CA, USA), mouse anti-NeuN (1:200, MAB377, Millipore, Burlington, MA, USA) and mouse anti-GFAP (1:200, Bio-Rad, USA). The sections were then incubated with secondary antibodies, including donkey anti-mouse IgG Highly cross-absorbed secondary antibody, Alexa Fluor 488 (1:1,000, A-21202, Invitrogen, Carlsbad, CA, USA) and donkey anti-rabbit IgG highly cross-absorbed secondary antibody, Alexa Fluor 555 (1:1,000, A-31572, Invitrogen, Carlsbad, CA, USA) for 1 h at room temperature. Finally, the slides were rinsed with PBS three times and counterstained with fluoroshield mounting medium with DAPI (ab104139, Abcam, Burlingame, CA, USA) for 10 min. All images were observed under a laser confocal microscope (Leica DMi8, Germany).

### Fluoro-Jade B (FJB) Staining

Fluoro-Jade B (FJB) staining was performed following the manufactures’s manual (AG310, Millipore, Burlington, MA, USA). Frozen spinal cord sections were incubated with 1% sodium hydroxide in 80% alcohol for 5 min and 70% alcohol for 2 min, subsequently transferred to 0.06% potassium permanganate for 10 min. Afterward, sections were immersed in a 0.0004% fluoro-jade dye solution which contains 0.1% acetic acid for 20 min and washed by deionized water. Then, sections were dried at 50°C for 8 min in an oven. Afterward, the sections were immersed in the xylene for at least 1 min finally mounted with neutral balsam (G8590, Solarbio, Beijing, China). The sections were observed under a laser confocal microscope (Leica DMi8, Germany), and the images were captured by LASX software (Leica Microsystems, Wetzlar, Germany).

### TUNEL Staining

TUNEL staining was used to assess cell apoptotic status in spinal cord tissues. The spinal sections were stained by using the Roche *in situ* Cell Death Detection Kit (11684817910, Roche, Germany) according to the manufactures’ manual. After allowing to air dry, they were then sealed with DAPI. All sections were observed under a laser confocal microscope (Leica DMi8, Germany) and images were captured using LASX software.

### Enzyme-Linked Immunosorbent Assay (ELISA)

The levels of superoxidase dismutase (SOD) and glutathione peroxidase (GPx) in spinal cord tissue samples were estimated by using commercially available ELISA kits specific for rats (ab65354, Abcam, Burlingame, CA, USA; ab102530, Abcam, Burlingame, CA, USA) according to the manufacturer’s instructions. The absorbance was measured using an ELISA reader at OD450 nm and OD340 nm.

### Statistical Analysis

All data are expressed as the mean ± standard deviation and were analyzed using SPSS software (version 18, SPSS Inc., Chicago, IL, USA). Histopathology volume percentage data were performed using a one-way analysis of variance (ANOVA). Behavioral data were analyzed by two-way ANOVA followed by Tukey’s *post hoc* test. *P* < 0.05 was considered to be a significant difference.

## Results

### Treatment With 2-BFI Promotes Functional Recovery After SCI

**Figure 2:**
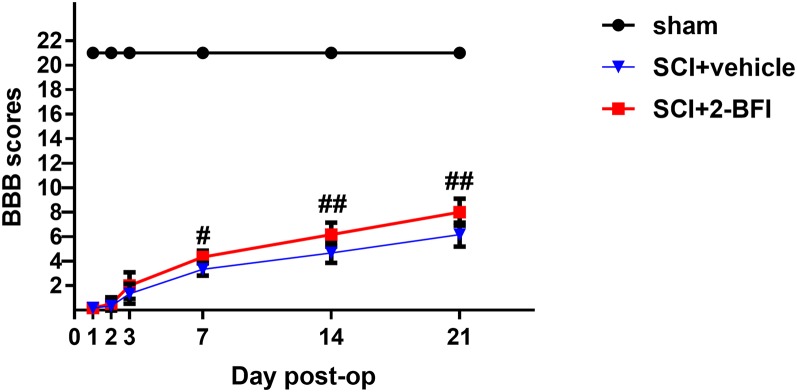
The locomotor recovery test after SCI was evaluated by the BBB locomotor rating scale. Although the 2-BFI treatment improves functional recovery (BBB scores) at 3 days after SCI, no significant differences were demonstrated. However, compared with the SCI+vehicle group, the BBB scores were significantly increased in the SCI+2-BFI group at 7, 14 and 21 days after SCI. Values are the mean ± SD. ^#^*P* < 0.05 vs. the SCI+vehicle group****; ^##^*P* < 0.01 vs. the SCI+vehicle group.

The locomotor recovery test after SCI was evaluated by the BBB locomotor rating scale. Normal scores (21 points) were observed in the sham group. One day after SCI, the BBB locomotor rating scale score was decreased promptly in both the vehicle group and the 2-BFI group. There was no significant difference between these two groups in BBB scores, even 3 days after SCI (Figure 2). However, the BBB scores were still higher in the 2-BFI group than in the vehicle group at 3 days after SCI, but the difference was not significant (2.00 ± 1.09 vs. 1.33 ± 0.82, *P* > 0.05). As shown in Figure 2, the average BBB score was significantly improved in rats treated with 2-BFI at both 7 days and 14 days after SCI (3.33 ± 0.52 vs. 4.33 ± 0.52, *P* < 0.05; 4.67 ± 0.82 vs. 6.1c7 ± 0.98, *P* < 0.05, respectively). Moreover, these differences became more and more remarkable at 21 days after SCI (6.17 ± 0.98 vs. 8.00 ± 1.10, *P* < 0.01). This result suggests that 2-BFI may have a potential protective effect on SCI.

### Treatment With 2-BFI Activates the Nrf2/HO-1 Signaling Pathway After SCI

**Figure 3:**
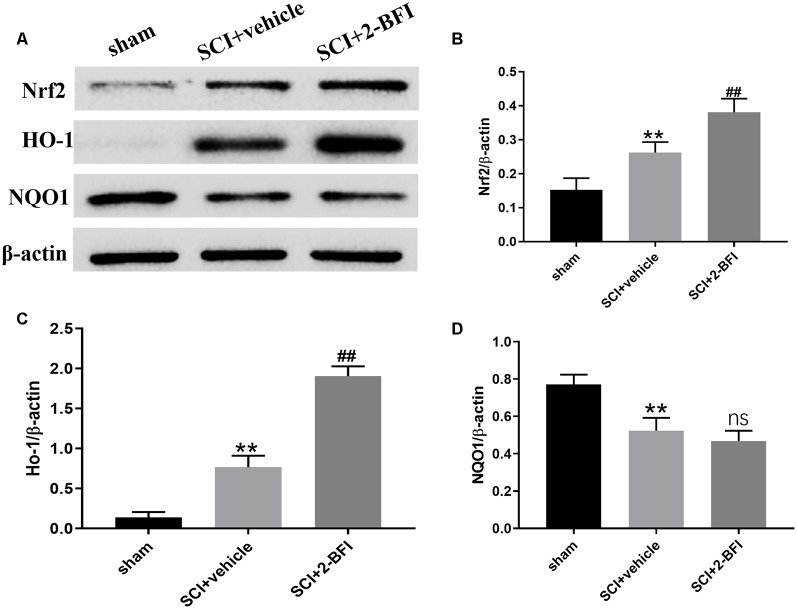
Expression profiles of the Nrf2, HO-1 and NQO1 proteins in the spinal cord tissue surrounding the damage of each group at 3 days after SCI **(A)**. Western blotting was performed to determine the expression profiles and quantification results of Nrf2 **(B)**, HO-1 **(C)** and NQO1** (D)**. Band density was quantified using ImageJ. *n* = 6 animals per group. Columns represent the mean ± SD. **P < 0.01 vs. the sham group; ^##^*P* < 0.01 vs. the SCI+vehicle group; ns, no significance vs. the SCI+vehicle group.

To investigate the potential role of 2-BFI in SCI models, we performed Western blotting to detect the expression of associated proteins, such as Nrf2, HO-1, and NQO1, after treatment with 2-BFI (Figure 3). As shown in Figures 3A–C, the Nrf2 and HO-1 protein levels increased significantly after SCI (*P* < 0.01). Additionally, Nrf2, as well as HO-1, was further increased significantly in the 2-BFI group compared with the vehicle group (*P* < 0.01; Figures 3A–C), whereas the NQO1 protein expression was decreased in the vehicle group compared with the sham group (*P* < 0.01; Figure 3D). Moreover, no significant difference was observed in the expression of NQO1 between the vehicle group and the 2-BFI group (*P* > 0.05).

### Treatment With 2-BFI Activates the Nrf2/HO-1 Signaling Pathway in Neurons and Astrocytes

**Figure 4:**
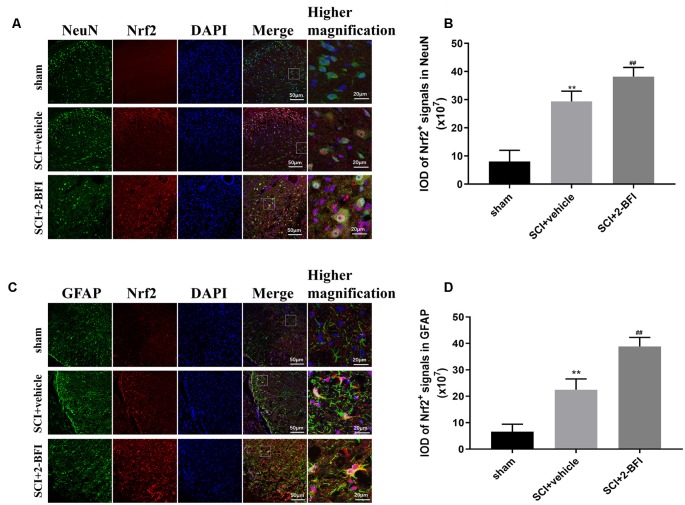
Double-immunofluorescence staining images of Nrf2 (red) with NeuN (green)-marked neurons **(A)** and Nrf2 (red) with GFAF (green)-marked astrocytes **(C)** to show the expression profiles in the sham group, vehicle group and 2-BFI group. Nuclei were counterstained with DAPI (blue) in the same view for each section. Immunofluorescence analysis was used to detect the IOD of Nrf2 signals in neurons **(B)** and astrocytes **(D)** at day 3 after the operation. Scale bars are 10 μm. Immunofluorescence intensities were determined using ImageJ software. *n* = 6 animals per group. Columns represent the mean ± SD. **P < 0.01 vs. the sham group; ^##^*P* < 0.01 vs. the SCI+vehicle group.

To confirm that 2-BFI activated the Nrf2/HO-1 signaling pathway, we performed immunofluorescence staining to evaluate the expression of Nrf2 in neurons and astrocytes (Figure 4). Nrf2 expression was dramatically higher in both neurons and astrocytes after SCI than that in the sham cgroup (*P* < 0.01; Figures 4A,C). In addition, the level of Nrf2 significantly increased in the SCI+2-BFI group than in the SCI+vehicle group (*P* < 0.01; Figures 4B,D).

### Treatment With 2-BFI Activates the Nrf2/HO-1 Signaling Pathway to Promote Antioxidant and Antiapoptotic Effects in SCI

**Figure 5:**
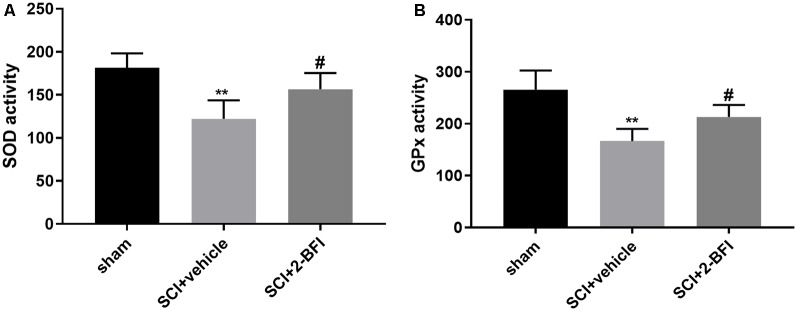
The levels of superoxidase dismutase (SOD) and glutathione peroxidase (GPx) in spinal cord tissue samples were estimated by ELISAs **(A,B)**. *n* = 6 animals per group. Columns represent the mean ± SD. **P < 0.01 vs. the sham group; ^#^*P* < 0.05 vs. the SCI+vehicle group.

To determine the local antioxidative levels, we detected the SOD and GPx activities in the damaged spinal cord tissue. Compared with those in the sham group, the activities of both SOD and GPx were significantly decreased in the vehicle group (*P* < 0.01; Figures 5A,B). Meanwhile, SOD and GPx were significantly enhanced in the 2-BFI group (*P* < 0.05; Figures 5A,B). These interesting findings suggested that 2-BFI may enhance antioxidant enzyme activities to promote locomotor recovery after SCI.

**Figure 6:**
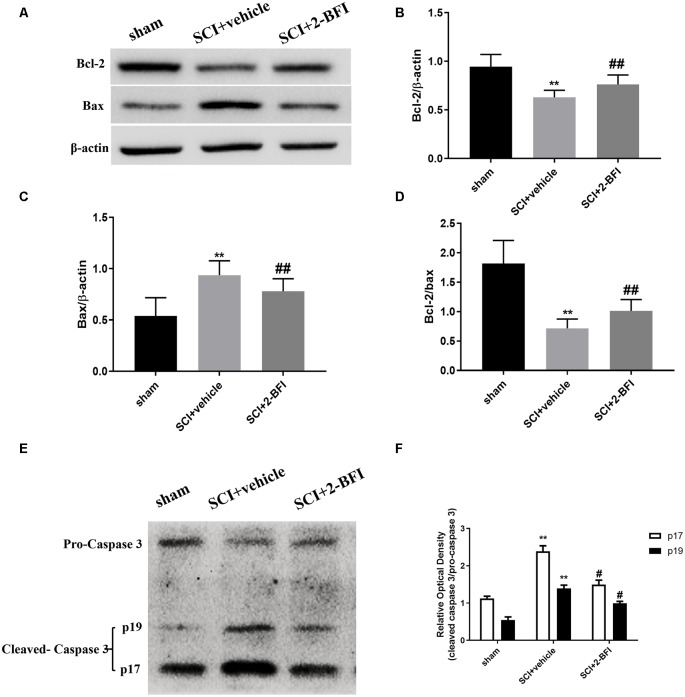
The expression levels of the Bcl-2, BAX, and caspase-3 proteins were detected using Western blotting at 3 days after SCI **(A,E)**. Quantification of the Bcl-2 **(B)**, BAX **(C)**, the ratio of Bcl-2/Bax **(D)** and the ratio of cleaved-caspase 3 (p17, p19)/pro-caspase 3 **(F)** verified the antiapoptotic effect of 2-BFI. Band density was quantified using ImageJ software. *n* = 6 animals per group. Columns represent the mean ± SD. **P < 0.01 vs. the sham group; ^##^*P* < 0.01 vs. the SCI+vehicle group; ^#^*P* < 0.05 vs. the SCI+vehicle group.

The expression levels of Bcl-2, Bax and caspase-3 including pro-caspase-3, the p19 caspase-3 subunit and the p17 caspase-3 subunit (which could be observed at 34 KDa, 19 KDa, and 17 KDa, respectively) were analyzed by Western blotting to further verify the antiapoptotic effect of 2-BFI in SCI (Figures 6A,E). The Bax and cleaved caspase-3 levels were appreciably increased in the spinal cord tissue collected from rats subjected to SCI, while the Bcl-2 protein levels were markedly reduced (*P* < 0.01 and *P* < 0.05, respectively; Figure 6). However, the application of 2-BFI significantly increased the Bcl-2 levels after SCI (*P* < 0.01; Figure 6B). In contrast, the Bax and cleaved caspase-3 levels were downregulated by 2-BFI (Figures 6C,F). Meanwhile, the Bcl-2/Bax ratio was effectively reduced after SCI and increased in the SCI+2-BFI group compared with the vehicle group (*P* < 0.01; Figure 6D).

**Figure 7:**
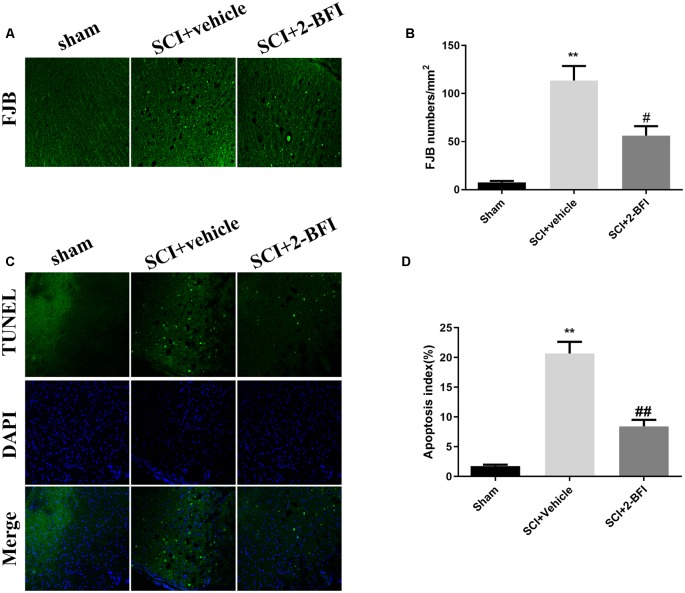
Evaluation of neuronal death at day 3 after operation. Neuronal death was examined by FJB staining **(A)**. FJB-positive (green) cells per mm^2^ were quantified accordingly **(B)**. Neuronal apoptosis was examined by TUNEL staining **(C)**. TUNEL-positive (green) cells per mm^2^ were quantified accordingly **(D)**. Scale bars are 10 μm. The number of positive cells was determined using ImageJ software. *n* = 6 animals per group. Columns represent the mean ± SD. **P < 0.01 vs. the sham group; ^##^*P* < 0.01 vs. the SCI+vehicle group; ^#^*P* < 0.05 vs. the SCI+vehicle group.

The effect of 2-BFI on cell necrosis and apoptosis was evaluated by FJB staining and TUNEL staining (Figures 7A,C). There were seldom FJB-positive or TUNE-positive cells in the spinal cord of the sham group. Obviously, a number of FJB-positive and TUNE-positive cells were observed in the SCI+vehicle group. The treatment of 2-BFI significantly reduced the necrosis and apoptosis (*P* < 0.05 and *P* < 0.01, respectively; Figures 7B,D).

## Discussion

SCI is among the most serious conditions that induce dysfunction of the limbs and trunk worldwide (Hachem et al., 2017). Although there is no definite treatment for SCI, various animal models are involved in experimental treatment to explore any potential therapies. Among these animal species, rat models were the most widely used for in-depth research of physiological and pathological events (Sharif-Alhoseini et al., 2017). According to the studies by Gris et al. (2004) and Hurtado et al. (2012), SCI models performing double laminectomies (Th10-Th11) yielded poorer BBB scores than when a single laminectomy (Th11) was done to expose the spinal cord. We were tended to follow the method as mention above to make the SCI model, however, the visual field of the model was not clear enough. Then we decided to extend another segment caudally. This present study was design to provide new evidence regarding the mechanism underlying the neuroprotective effect of 2-BFI in a rat model of SCI with laminectomies (Th10-Th12). Such neuroprotection by 2-BFI was most likely accomplished by activating the Nrf2/HO-1 pathway, which protected neurons by increasing the antioxidant activities and suppressed neuronal apoptosis by regulating the antioxidative injury and blocking apoptosis.

Oxidative injury and apoptosis play the most important roles in central nervous system neurological disorders, including SCI (Liu and Xu, 2012). Reactive oxygen species (ROS), such as hydrogen peroxide, superoxide, and hydroxyl radicals, increased significantly within hours after SCI (Choi et al., 2015; Zhang et al., 2016). Among the antioxidative mechanisms, one of the major adaptive responses in the central nervous system is mediated by Nrf2. As shown in the present study, the level of Nrf2 expression increased significantly after SCI, which was according to the previous research. In addition, Nrf2 may play an important role in defense against oxidative stress possibly by activation of the cellular antioxidant machinery as well as suppression of pro-inflammatory signaling pathways (Li et al., 2008).

Normal cellular functions are controlled by cells by maintaining the oxidant and antioxidant balance. In response to oxidative stress, Nrf2 induces not only the expression of a number of antioxidant genes but also the activity of various antioxidant enzymes, such as HO-1, SOD, and GPx (Wang et al., 2012; Lin et al., 2017; Wei et al., 2018). As an indicator and regulator of oxidative stress, the Nrf2/HO-1 signaling pathway has been demonstrated to play a substantial role in protecting against oxidative stress for many years (Gan and Johnson, 2014; Freitas et al., 2015; Lu et al., 2018; Wei et al., 2018). A previous study showed that SCI induces increased expression of Nrf2 in neurons and astrocytes as early as 30 min following SCI, and the levels remained elevated for 3 days (Wang et al., 2012). Simultaneously, the expression of HO-1 increased significantly from 1 day after SCI and continued to increase by 3 days post-injury, whereas the levels of NQO1 peaked at 30 min immediately after SCI and gradually decreased until they eventually showed no significant change after 3 days (Wang et al., 2012). Activation of the Nrf2 signaling pathway following SCI regulates genes involved in antioxidant defenses, which significantly increase the expression of the antioxidants HO-1 and NQO1 (Zhou et al., 2017; Wei et al., 2018). In the present study, the upregulated expression of Nrf2 after SCI was observed as well as the expression of HO-1. However, the expression of NQO1 does not seem to be fully consistent with that of previous studies due to the tissue collection method. Moreover, 2-BFI further increased the expression of Nrf2 and HO-1, which indicates that the activation of Nrf2/HO-1 signaling by 2-BFI protects neurons from damage after SCI in the present study. The present study observed a significant reduction in the activities of the enzymatic antioxidants after SCI, including SOD and GPx, which protect against cellular damage by diminishing oxidative toxicity. Interestingly, the activities of SOD and GPx were upgraded by treatment with 2-BFI after SCI, which was consistent with the effect of 2-BFI in Alzheimer’s disease (Tian et al., 2017). Taken together, these findings indicated that 2-BFI may attenuate SCI by inhibiting oxidative stress and neuronal apoptosis to improve functional recovery after SCI *via* activation of the Nrf2 signaling pathway.

Apoptosis is an active process of programmed cell death, which is regulated by various genes including the Bcl-2 family, caspase family and cancer genes (Edlich, 2018). SCI-induced apoptosis leads to neuronal cell death in the spinal cord, further resulting in neuronal dysfunction from 6 h to 3 weeks after injury (Li et al., 2015). As demonstrated in previous studies, 2-BFI showed potent neuroprotective effects by attenuating neural apoptosis through upregulating the neuroprotective gene Bcl-2 in transient cerebral ischemia rats (Han et al., 2010). Meanwhile, 2-BFI improved the impairments by attenuating the expression of the apoptotic factors in Alzheimer’s disease rats (Tian et al., 2017). Consistent with these studies, we found that 2-BFI upregulated the expression of Bcl-2 and downregulated the expression of Bax and cleaved caspase 3 (both p17 and p19), further confirming the neuroprotective effects. These results indicate that the antiapoptotic effect of 2-BFI may contribute to its neuroprotective effect in SCI rats. Moreover, the decreased level of necrosis and apoptosis estimated by FJB staining and TUNEL staining showed corroborative evidence of neuroprotection of 2-BFI.

It is interesting to note that the cleaved caspase 3 was comparable in the sham group and SCI+2-BFI group as shown in Figure 6, which demonstrated the cleaved caspase 3 might indicate something else, such as reactive astrogliosis and the infiltration of macrophages (Wagner et al., 2011).

Several limitations of the present study should be mentioned here. First, because the molecular nature of the I_2_ receptors is elusive (Li, 2017), the precise molecular mechanism underlying the activation of Nrf2 by 2-BFI has yet to be identified. Second, our study did not include any experiment about blockage of Nrf2, hence, we cannot exclude the possibility that the inhibited inflammatory effects or other effects of 2-BFI also play roles in the neuroprotection after SCI. Therefore, these issues will be examined in our future studies.

In conclusion, our observations indicated that 2-BFI may attenuate SCI *via* the Nrf2/HO-1 signaling pathway by inhibiting oxidative stress and neuronal apoptosis. This study provides new information on the neuroprotective effect of 2-BFI following SCI and will help improve our understanding of imidazoline I_2_ receptor pharmacology.

## Data Availability Statement

The raw data supporting the conclusions of this article will be made available by the authors, without undue reservation, to any qualified researcher.

## Ethics Statement

The animal study was reviewed and approved by Animal Care and Use Committee of Soochow University.

## Author Contributions

XL and JZ conceived the study and design, contributed equally. JZ, QR, HN, and WS analyzed and interpreted the data. XL and JZ drafted the main manuscript text. DL, HY, and GC performed critical revision of the manuscript.

## Conflict of Interest

The authors declare that the research was conducted in the absence of any commercial or financial relationships that could be construed as a potential conflict of interest.

## References

[B1] Basso D. M. Beattie M. S. Bresnahan J. C. (1995). A sensitive and reliable locomotor rating scale for open field testing in rats. J. Neurotrauma 12, 1–21. 10.1089/neu.1995.12.1 7783230

[B2] Basso D. M. Fisher L. C. Anderson A. J. Jakeman L. B. McTigue D. M. Popovich P. G. (2006). Basso Mouse Scale for locomotion detects differences in recovery after spinal cord injury in five common mouse strains. J. Neurotrauma 23, 635–659. 10.1089/neu.2006.23.635 16689667

[B3] Can H. Aydoseli A. Gömleksiz C. Göker B. Altunrende M. E. Dolgun M.. (2017). Combined and individual use of pancaspase inhibitor Q-VD-OPh and NMDA receptor antagonist riluzole in experimental spinal cord injury. Ulus. Travma Acil Cerrahi Derg. 23, 452–458. 10.5505/tjtes.2017.09694 29115658

[B4] Chen S. Ye J. Chen X. Shi J. Wu W. Lin W.. (2018). Valproic acid attenuates traumatic spinal cord injury-induced inflammation *via* STAT1 and NF-kappaB pathway dependent of HDAC3. J. Neuroinflammation 15:150. 10.1186/s12974-018-1193-6 29776446 PMC5960086

[B5] Choi E. K. Yeo J. S. Park C. Y. Na H. Lim J. Lee J. E.. (2015). Inhibition of reactive oxygen species downregulates the MAPK pathway in rat spinal cord after limb ischemia reperfusion injury. Int. J. Surg. 22, 74–78. 10.1016/j.ijsu.2015.08.016 26283297

[B6] Edlich F. (2018). BCL-2 proteins and apoptosis: recent insights and unknowns. Biochem. Biophys. Res. Commun. 500, 26–34. 10.1016/j.bbrc.2017.06.190 28676391

[B7] Freitas A. E. Egea J. Buendía I. Navarro E. Rada P. Cuadrado A.. (2015). Agmatine induces Nrf2 and protects against corticosterone effects in hippocampal neuronal cell line. Mol. Neurobiol. 51, 1504–1519. 10.1007/s12035-014-8827-1 25084759

[B8] Gan L. Johnson J. A. (2014). Oxidative damage and the Nrf2-ARE pathway in neurodegenerative diseases. Biochim. Biophys. Acta 1842, 1208–1218. 10.1016/j.bbadis.2013.12.011 24382478

[B9] Gris D. Marsh D. R. Oatway M. A. Chen Y. Hamilton E. F. Dekaban G. A.. (2004). Transient blockade of the CD11d/CD18 integrin reduces secondary damage after spinal cord injury, improving sensory, autonomic, and motor function. J. Neurosci. 24, 4043–4051. 10.1523/JNEUROSCI.5343-03.2004 15102919 PMC6729422

[B10] Hachem L. D. Ahuja C. S. Fehlings M. G. (2017). Assessment and management of acute spinal cord injury: from point of injury to rehabilitation. J. Spinal Cord Med. 40, 665–675. 10.1080/10790268.2017.1329076 28571527 PMC5778930

[B11] Han Z. Xiao M. J. Shao B. Zheng R. Y. Yang G. Y. Jin K. (2009). Attenuation of ischemia-induced rat brain injury by 2-(-2-benzofuranyl)-2-imidazoline, a high selectivity ligand for imidazoline I_2_ receptors. Neurol. Res. 31, 390–395. 10.1179/174313209x444116 19508825

[B12] Han Z. Zhang H. X. Tian J. S. Zheng R. Y. Hou S. T. (2010). 2-(-2-benzofuranyl)-2-imidazoline induces Bcl-2 expression and provides neuroprotection against transient cerebral ischemia in rats. Brain Res. 1361, 86–92. 10.1016/j.brainres.2010.09.029 20840843

[B13] Huo J. Ma R. Chai X. Liang H. J. Jiang P. Zhu X. L.. (2019). Inhibiting a spinal cord signaling pathway protects against ischemia injury in rats. J. Thorac. Cardiovasc. Surg. 157, 494.e1–503.e1. 10.1016/j.jtcvs.2018.07.045 30195603

[B14] Hurtado A. Marcillo A. Frydel B. Bunge M. B. Bramlett H. M. Dietrich W. D. (2012). Anti-CD11d monoclonal antibody treatment for rat spinal cord compression injury. Exp. Neurol. 233, 606–611. 10.1016/j.expneurol.2010.11.015 21145887 PMC3080438

[B15] Jiang S. X. Zheng R. Y. Zeng J. Q. Li X. L. Han Z. Hou S. T. (2010). Reversible inhibition of intracellular calcium influx through NMDA receptors by imidazoline I_2_ receptor antagonists. Eur. J. Pharmacol. 629, 12–19. 10.1016/j.ejphar.2009.11.063 19958763

[B16] Lee S. H. Kim Y. Rhew D. Kim A. Jo K. R. Yoon Y.. (2017). Effect of canine mesenchymal stromal cells overexpressing heme oxygenase-1 in spinal cord injury. J. Vet. Sci. 18, 377–386. 10.4142/jvs.2017.18.3.377 27586469 PMC5639091

[B18] Li J. X. (2017). Imidazoline I_2_ receptors: an update. Pharmacol. Ther. 178, 48–56. 10.1016/j.pharmthera.2017.03.009 28322973 PMC5600648

[B17] Li G. Jia Z. Cao Y. Wang Y. Li H. Zhang Z.. (2015). Mitochondrial division inhibitor 1 ameliorates mitochondrial injury, apoptosis, and motor dysfunction after acute spinal cord injury in rats. Neurochem. Res. 40, 1379–1392. 10.1007/s11064-015-1604-3 25968480

[B19] Li W. Khor T. O. Xu C. Shen G. Jeong W. S. Yu S.. (2008). Activation of Nrf2-antioxidant signaling attenuates NFkappaB-inflammatory response and elicits apoptosis. Biochem. Pharmacol. 76, 1485–1489. 10.1016/j.bcp.2008.07.017 18694732 PMC2610259

[B22] Lin Y. Vreman H. J. Wong R. J. Tjoa T. Yamauchi T. Noble-Haeusslein L. J. (2007). Heme oxygenase-1 stabilizes the blood-spinal cord barrier and limits oxidative stress and white matter damage in the acutely injured murine spinal cord. J. Cereb. Blood Flow Metab. 27, 1010–1021. 10.1038/sj.jcbfm.9600412 17047682

[B21] Lin W. Wang S. Yang Z. Lin J. Ke Q. Lan W.. (2017). Heme oxygenase-1 inhibits neuronal apoptosis in spinal cord injury through down-regulation of Cdc42-MLK3-MKK7-JNK3 axis. J. Neurotrauma 34, 695–706. 10.1089/neu.2016.4608 27526795

[B20] Lin W. P. Xiong G. P. Lin Q. Chen X. W. Zhang L. Q. Shi J. X.. (2016). Heme oxygenase-1 promotes neuron survival through down-regulation of neuronal NLRP1 expression after spinal cord injury. J. Neuroinflammation 13:52. 10.1186/s12974-016-0521-y 26925775 PMC4772494

[B23] Liu N. K. Xu X. M. (2012). Neuroprotection and its molecular mechanism following spinal cord injury. Neural Regen. Res. 7, 2051–2062. 10.3969/j.issn.1673-5374.2012.26.007 25624837 PMC4296426

[B24] Lu T. Wu X. Wei N. Liu X. Zhou Y. Shang C.. (2018). Lipoxin A4 protects against spinal cord injury *via* regulating Akt/nuclear factor (erythroid-derived 2)-like 2/heme oxygenase-1 signaling. Biomed. Pharmacother. 97, 905–910. 10.1016/j.biopha.2017.10.092 29136768

[B25] Meng H. Y. Shao D. C. Li H. Huang X. D. Yang G. Xu B.. (2018). Resveratrol improves neurological outcome and neuroinflammation following spinal cord injury through enhancing autophagy involving the AMPK/mTOR pathway. Mol. Med. Rep. 18, 2237–2244. 10.3892/mmr.2018.9194 29956767

[B26] Ni H. Rui Q. Lin X. Li D. Liu H. Chen G. (2019). 2-BFI provides neuroprotection against inflammation and necroptosis in a rat model of traumatic brain injury. Front. Neurosci. 13:674. 10.3389/fnins.2019.00674 31293382 PMC6606784

[B27] Niture S. K. Kaspar J. W. Shen J. Jaiswal A. K. (2010). Nrf2 signaling and cell survival. Toxicol. Appl. Pharmacol. 244, 37–42. 10.1016/j.taap.2009.06.009 19538984 PMC2837794

[B28] Sampson C. Zhang Y. Del Bello F. Li J. X. (2012). Effects of imidazoline I2 receptor ligands on acute nociception in rats. Neuroreport 23, 73–77. 10.1097/wnr.0b013e32834e7db3 22107843

[B29] Sharif-Alhoseini M. Khormali M. Rezaei M. Safdarian M. Hajighadery A. Khalatbari M. M.. (2017). Animal models of spinal cord injury: a systematic review. Spinal Cord 55, 714–721. 10.1038/sc.2016.187 28117332

[B30] Soubeyrand M. Badner A. Vawda R. Chung Y. S. Fehlings M. G. (2014). Very high resolution ultrasound imaging for real-time quantitative visualization of vascular disruption after spinal cord injury. J. Neurotrauma 31, 1767–1775. 10.1089/neu.2013.3319 24831774 PMC4186763

[B31] Tian J. S. Zhai Q. J. Zhao Y. Chen R. Zhao L. D. (2017). 2–(2-benzofuranyl)-2-imidazoline (2-BFI) improved the impairments in AD rat models by inhibiting oxidative stress, inflammation and apoptosis. J. Integr. Neurosci. 16, 385–400. 10.3233/JIN-170032 28891528

[B32] Wagner D. C. Riegelsberger U. M. Michalk S. Härtig W. Kranz A. Boltze J. (2011). Cleaved caspase-3 expression after experimental stroke exhibits different phenotypes and is predominantly non-apoptotic. Brain Res. 1381, 237–242. 10.1016/j.brainres.2011.01.041 21256117

[B34] Wang X. Campos C. R. Peart J. C. Smith L. K. Boni J. L. Cannon R. E.. (2014). Nrf2 upregulates ATP binding cassette transporter expression and activity at the blood-brain and blood-spinal cord barriers. J. Neurosci. 34, 8585–8593. 10.1523/JNEUROSCI.2935-13.2014 24948812 PMC4061395

[B35] Wang X. de Rivero Vaccari J. P. Wang H. Diaz P. German R. Marcillo A. E.. (2012). Activation of the nuclear factor E2-related factor 2/antioxidant response element pathway is neuroprotective after spinal cord injury. J. Neurotrauma 29, 936–945. 10.1089/neu.2011.1922 21806470 PMC3303102

[B33] Wang P. Wang Z. W. Lin F. H. Han Z. Hou S. T. Zheng R. Y. (2011). 2-BFI attenuates experimental autoimmune encephalomyelitis-induced spinal cord injury with enhanced B-CK, CaATPase, but reduced calpain activity. Biochem. Biophys. Res. Commun. 406, 152–157. 10.1016/j.bbrc.2011.02.018 21303658

[B36] Wei W. Shurui C. Zipeng Z. Hongliang D. Hongyu W. Yuanlong L.. (2018). Aspirin suppresses neuronal apoptosis, reduces tissue inflammation, and restrains astrocyte activation by activating the Nrf2/HO-1 signaling pathway. Neuroreport 29, 524–531. 10.1097/wnr.0000000000000969 29381509

[B37] Zhang B. Bailey W. M. McVicar A. L. Gensel J. C. (2016). Age increases reactive oxygen species production in macrophages and potentiates oxidative damage after spinal cord injury. Neurobiol. Aging 47, 157–167. 10.1016/j.neurobiolaging.2016.07.029 27596335 PMC5075497

[B38] Zhou Z. Liu C. Chen S. Zhao H. Zhou K. Wang W.. (2017). Activation of the Nrf2/ARE signaling pathway by probucol contributes to inhibiting inflammation and neuronal apoptosis after spinal cord injury. Oncotarget 8, 52078–52093. 10.18632/oncotarget.19107 28881715 PMC5581014

